# Secondary Metabolites from *Dendrobium nobile* and Their Activities Induce Metabolites Apoptosis in OSC-19 Cells

**DOI:** 10.3390/molecules28083423

**Published:** 2023-04-13

**Authors:** Yufan Meng, Maosheng Zhang, Yike Fang, Jianwen Yang, Minjian Dong, Chengxin Sun, Shiji Xiao

**Affiliations:** 1 Key Laboratory of Basic Pharmacology of Guizhou Province and School of Pharmacy, Zunyi Medical University, Zunyi 563000, China; m19197841591@163.com (Y.M.);; 2 Key Laboratory of Basic Pharmacology of Ministry of Education and Joint International Research Laboratory of Ethnomedicine of Ministry of Education, Zunyi Medical University, Zunyi 563000, China

**Keywords:** *Dendrobium nobile*, moscatilin, apoptosis, MAPK

## Abstract

To identify potential drug candidates, secondary metabolites of *Dendrobium nobile* were performed. As a result, two previously undescribed phenanthrene derivatives with a spirolactone ring (**1** and **2**), along with four known compounds, *N*-*trans*-cinnamoyltyramine (**3**), *N*-*trans*-*p*-coumaroyltyramine (**4**), *N*-*trans*-feruloyltyramine (**5**), and moscatilin (**6**), were isolated from *Dendrobium nobile*. The structures of the undescribed compounds were elucidated using NMR spectroscopy, electronic circular dichroism (ECD) calculations, and extensive spectroscopic data analysis. The cytotoxic effects of compounds on human tongue squamous cells OSC-19 were determined using MTT at concentrations of 2.5 μM, 5 μM, 10 μM, and 20 μM. Compound **6** exhibited potent inhibitory activity against OSC-19 cells with an IC_50_ of 1.32 μM. Migration assays and western blot assays demonstrated that compound **6** effectively inhibited migration by down-regulating MMP2 and MMP9 at concentrations of 0.5 μM and 1 μM. To investigate its effect on apoptosis, we performed AO/PI staining, flow cytometry, and WB experiments. The results showed that increasing concentrations led to increased red fluorescence, decreased green fluorescence, increased apoptosis rate, decreased expression of bcl-2, caspase 3, caspase 9, and parp proteins, and increased bax expression. Furthermore, the phosphorylation of JNK and P38 was activated, suggesting that compound **6** may induce apoptosis via the MAPK pathway.

## 1. Introduction

Tongue cancer is a highly malignant tumor that occurs in the oral and maxillofacial region. It has the highest incidence rate among all oral cancers, accounting for approximately one-third of cases [1,2]. This type of cancer is characterized by rapid growth, strong infiltration, and high malignancy, which can result in impaired speech, mastication, and respiratory function, posing a significant threat to human health [3,4,5]. Searching for natural anticancer remedies with low toxicity and high efficacy has become a topic of great interest for researchers both at home and abroad. One such plant under investigation is *Dendrobium nobile* Lindl, a genus of the family orchidaceae, which is primarily distributed in China (specifically, Sichuan, Yunnan, Guizhou, Taiwan, Hu-bei, Guangdong, Hainan, and Guangxi provinces), and grows on trees in forest slopes and on rocks by the roadside at altitudes ranging from 800 to 1700 m above sea level. *D. nobile* has been used for thousands of years as a traditional Chinese herbal medicine and is also consumed as a food [6]. Previous studies have identified bibenzyls [7,8], sesquiterpenes [9,10,11], alkaloids [12,13], and phenanthrenes [8] as the primary components of *D. nobile*. Pharmacological research has revealed that *D. nobile* exhibits numerous health benefits, including immunomodulating, neuroprotective, hepatoprotective, anti-tumor, anti-oxidation, anti-diabetic, anti-platelet aggregation, and bacteriostasis activities. Previous studies have demonstrated that bibenzyls and their derivatives possess outstanding anti-tumor effects in vitro [14,15]. In our continued efforts to discover new and distinctive bioactive compounds from the *Dendrobium* genus, we have isolated two previously unknown phenanthrene derivatives featuring a spirolactone ring (**1** and **2**), along with four known compounds (**3**–**6**), from *D. nobile*. The structures of these previously unknown compounds were determined using NMR spectroscopy, electronic circular dichroism (ECD) calculations, and extensive spectroscopic data analysis. We evaluated the cytotoxic effects of the isolated compounds on the OSC-19 human tongue squamous cell line using the MTT assay, finding that compound **6** showed excellent inhibitory activity against this cell line. Furthermore, we investigated its anticancer mechanism.

## 2. Results and Discussion

### 2.1. Isolation and Structure Elucidation

**Figure 1:**
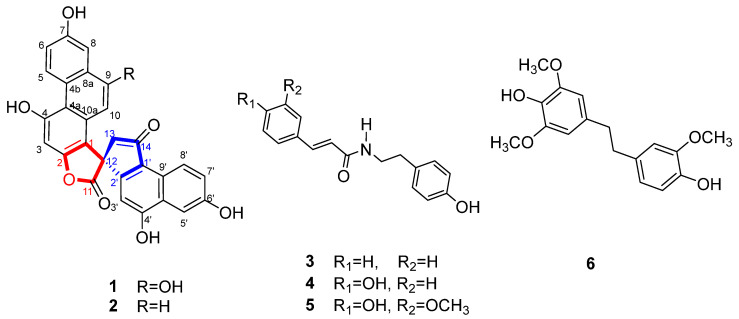
Structures of compounds **1**–**6**.

The dried *D. nobile* was refluxed with 90% methanol to obtain a crude extract. The EtOAc extract was separated by various column chromatographers to obtain compounds **1**–**6** (Figure 1). 

**Figure 2:**
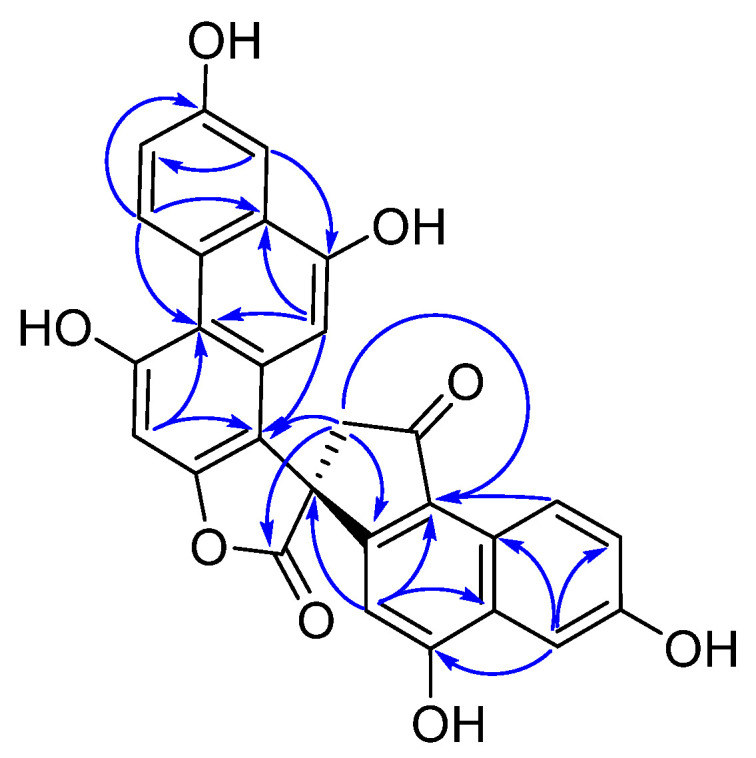
Selected HMBC correlations of **1**. The blue arrows represent the nuclear magnetic relationship between two atoms.

**Figure 3:**
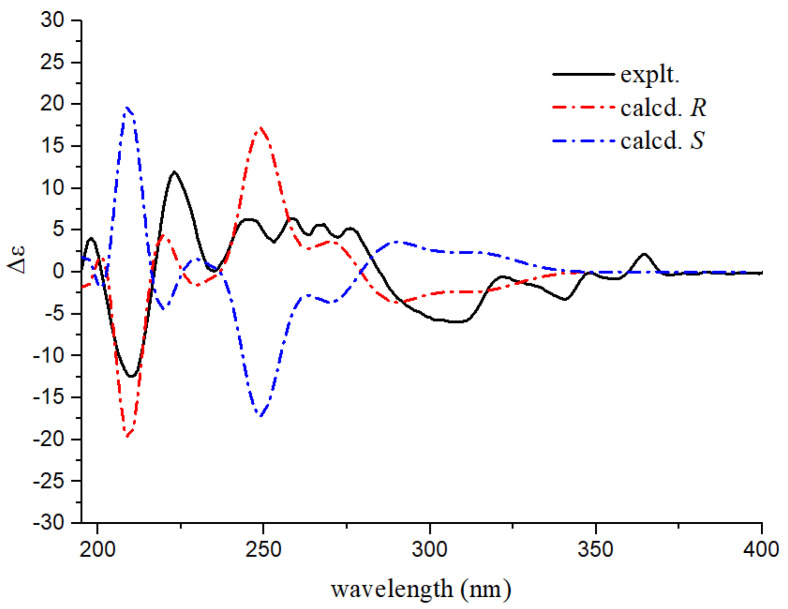
Calculated ECD spectra and experimental curve of **1**.

Compound **1** was obtained as a white powder. Its molecular formula, C_28_H_16_O_8_, was established from the *quasi*-molecular ion peak at *m*/*z* 481.1026 [M + H]^+^ (calcd. for C_28_H_17_O_8_^+^: 481.0923) and *m*/*z* 479.0517 [M − H]^−^ (calcd. for C_28_H_15_O_8_^−^: 479.0767) in the HR-ESIMS, which indicated 21 degrees of unsaturation. The ^1^H NMR (Table 1) and ^1^H-^1^H COSY spectra of **1** showed two ABX aromatic rings at *δ*_H_ 7.24 (1H, dd, *J* = 9.4, 2.8 Hz), 7.68 (1H, d, *J* = 2.8 Hz), 9.68 (1H, d, *J* = 9.4 Hz), and *δ*_H_ 7.45 (1H, dd, *J* = 9.0, 2.6 Hz), 7.66 (1H, d, *J* = 2.6 Hz), 9.11 (1H, d, *J* = 9.0 Hz); three aromatic singlets at *δ*_H_ 7.01 (1H, s), 6.40 (1H, s), and 6.39 (1H, s); and a pair of methylene protons at *δ*_H_ 3.23 (1H, d, *J* = 18.5 Hz) and 3.36 (1H, d, *J* = 18.5 Hz). The ^13^C NMR and HSQC spectra of compound **1** showed 28 signals, including a carbonyl group at *δ*_C_ 201.1, an ester group at *δ*_C_ 179.3, a methylene carbon at *δ*_C_ 47.9, a quaternary carbon at *δ*_C_ 54.1, and 22 aromatic carbon signals at *δ*_C_ 95.1–160.9. These spectroscopic features suggested that compound **1** was a phenanthrene derivative with a spirolactone ring [16]. The HMBC correlations (Figure 2) of H-8 to C-8a, C-9, C-4b, and H-5 to C-4b, C-4a, C-8a, C-7, suggested the existence of a hydroxyl group at C-7. The HMBC correlations of H-10 to C-1, C-4a, C-8a, C-9, and H-8 to C-9, established the presence of another hydroxyl group located at C-7. The HMBC correlations of H-3 to C-1, C-2, C-4, C-4a, in combination with the chemical shift values of C-3 (*δ*_C_ 95.3), C-4 (*δ*_C_ 152.3), and C-2 (*δ*_C_ 158.6), deduced the hydroxyl group affiliated to C-7, but an ester group connected with C-2. The HMBC correlations of H-13′ to C-1, C-2′, C-12′, C-1′, C-14′, displayed the quaternary carbon linked with C-1, C-2′, ester carbonyl group, and C-13′. The HMBC correlations of H-3′ to C-12′, C-1′, C-10′, and H-5′ to C-4′, indicated the existence of a hydroxyl group attached to C-4′. The HMBC correlations of H-5′ to C-4′, C-9′, C-7′, and H-8′ to C-1′, C-6′, supported the last hydroxyl group located at C-6′. Accordingly, the planar structure of compound **1** was established. The absolute configuration of compound **1** was finally established by calculated results from electronic circular dichroism (ECD) spectra using the time-dependent density functional theory (TD-DFT) method [17,18]. The calculated ECD spectra of the *R* matched well with the experimentally recorded ECD spectra (Figure 3). Therefore, the structure of compound **1** was established and named dendnobione A. 

Compound **2** was obtained as a white powder. The molecular formula of C_28_H_16_O_7_ was deduced using HR-ESIMS (*m*/*z* 465.0967 [M + H]^+^, calcd. for 465.0974 and *m*/*z* 463.0717 [M − H]^−^, calcd. for 463.0818), indicating 21 degrees of unsaturation. The ^1^H, ^13^C NMR, HSQC, and HMBC spectra (Supplementary Materials Figures S8–S12) were similar to those of compound **1**, which indicated that compound **1** was a homologue of compound **1**. The ^1^H NMR spectrum (Table 1) of compound **2** showed the presence of 10 aromatic proton signals, one signal more than compound **1**. By comparing the ^13^C NMR spectrum with those of compound **1**, the chemical shift of C-9 was shifted to high-field (*δ*_C_ 152.3 in **1**, *δ*_C_ 130.6 in **2**). Similarly, the signals of C-10 (*δ*_C_ 121.2), C-8a (*δ*_C_ 134.3), and C-8 (*δ*_C_ 112.5) were shifted (Δ*δ* = +20.9, +6.1, and +5.6, respectively). These changes indicated that the proton at C-9 in compound **2** replacement of the hydroxyl group at C-9 in compound **1**. The CD spectrum of compound **2** matched well with that of compound **1** and implied that the absolute configuration was *R*. In summary, the structure of compound **2** was determined and named dendnobione B.

Known compounds, *N*-*trans*-cinnamoyltyramine (compound **3**) [19], *N*-*trans*-*p*-coumaroyltyramine (compound **4**) [19], *N*-*trans*-feruloyltyramine (compound **5**) [20], and moscatilin (compound **6**) [21], were identified based on their identical NMR data to those in the literature.

### 2.2. Effect of Compounds on Proliferation Activity in OSC-19

**Figure 4:**
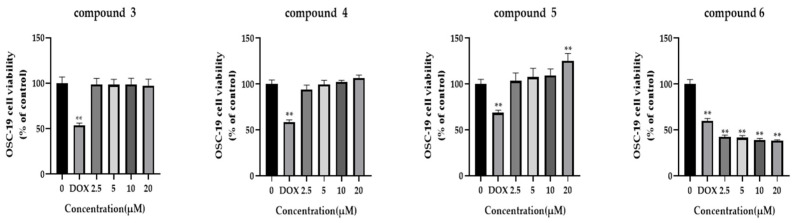
Cytotoxicity of moscatilin on OSC-19 cells. MTT assay was used to detect cell viability in OSC-19 cells after compound **3**–**6** exposure. n = 5, ** *p* < 0.01, compared with the 0 μM group.

In order to investigate the impact of various compounds on cellular proliferation in the OSC-19 cell line, an MTT assay was conducted at concentrations of 2.5 μM, 5 μM, 10 μM, and 20 μM. OSC-19 cells were exposed to varying concentrations of the compounds for 48 h. The results indicate that compounds **3**, **4**, and **5** exhibited insignificant inhibitory effects, whereas moscatilin demonstrated a dose-dependent inhibition of OSC-19 cell growth with a significant IC_50_ value of 1.32 μM (Figure 4).

### 2.3. Moscatilin Inhibits the Migration of Tongue Cancer Cells

**Figure 5:**
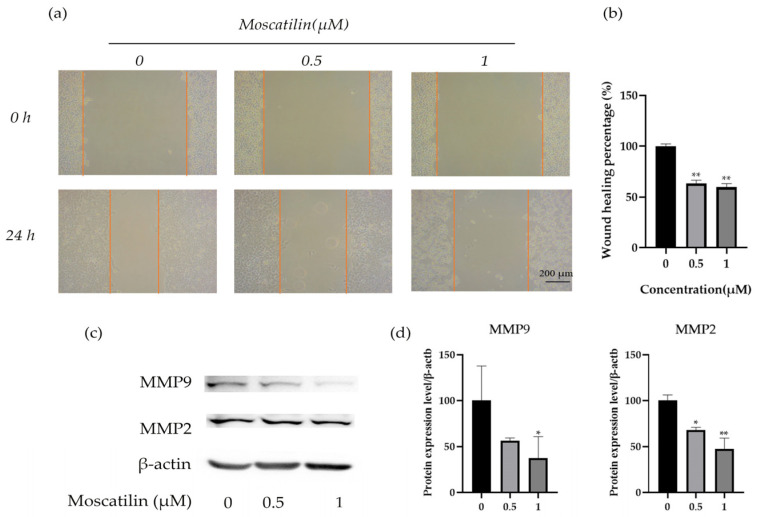
Effect of moscatilin on the migration of OSC-19 cells. (**a**) A wound healing assay was used to detect the migration of OSC-19 cells after moscatilin exposure. (**b**) Quantitative results were illustrated for the wound healing assay. * *p* < 0.05, ** *p* < 0.01 compared with the 0 μM group. (**c**) Western blotting assays were used to detect the protein levels of MMP2 and MMP9, and *β*-actin was used as a loading control. (**d**) Quantitative results were illustrated for the Western blot assay. * *p* < 0.05, ** *p* < 0.01 compared with 0 μM group.

Epithelial mesenchymal transition (EMT) is the process by which epithelial cells undergo transformation into mesenchymal cells. This process involves the loss of original cell polarity, leading to the breakdown of tight junctions and adhesive links between cells, resulting in infiltrative and wandering migration abilities. In addition, the expression of mesenchymal marker proteins, such as MMPs and vimentin is upregulated. When OSC-19 cells were treated with moscatilin for 24 h, their migration was found to be inhibited in a dose-dependent manner (Figure 5a,b). Furthermore, western blot analysis revealed a decreased expression of MMP2 and MMP9 (Figure 5c,d). These results suggest that moscatilin may suppress EMT by reducing the expression of MMP2 and MMP9 proteins, thereby inhibiting the migration of tongue cancer cells.

### 2.4. Apoptosis of Tongue Cancer Cells Induced by Moscatilin

**Figure 6:**
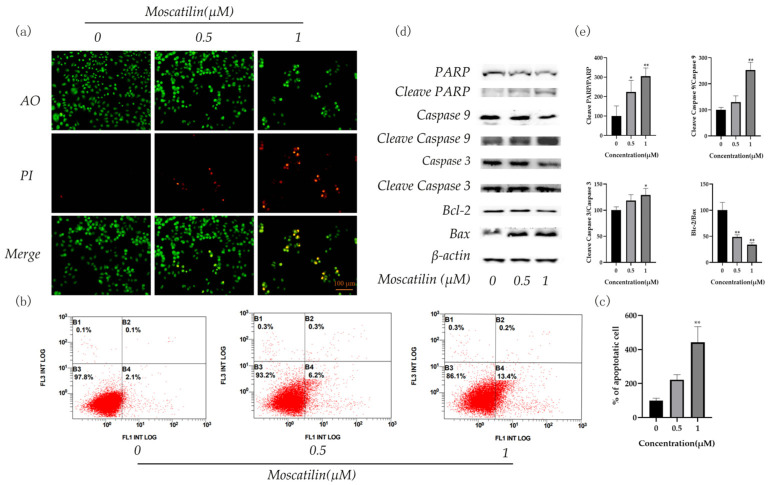
Moscatilin induces apoptosis in OSC-19 cells. (**a**) AO/PI staining assay was used to detect the effect of moscatilin on apoptosis. (**b**) Apoptosis of moscatilin-treated OSC-19 cells was determined using a flow cytometric assay. (**c**) The quantitative results were illustrated for apoptosis. ** *p* < 0.01 compare with 0 μM group. (**d**) Western blot assays were used to detect the protein levels of PARP, Cleaved PARP, Caspase 3, Cleaved Caspase 3, Caspase 9, Cleaved Caspase 9, Bax, and Bcl-2, and *β*-actin was used as a loading control. (**e**) The quantitative results were illustrated for the Western blot assay. * *p* < 0.05, ** *p* < 0.01 compared with the 0 μM group.

Figure 6 shows that the exposure of OSC-19 cells to moscatilin for 24 h resulted in chromatin shrinkage and condensation, reduced tumor-cell count, and increased red fluorescence in a dose-dependent manner (Figure 6a). Flow cytometry (FCM) analysis revealed that the apoptosis rate increased from 2.53 ± 0.28% and 5.63 ± 0.61% to 11.2 ± 1.92% after 24 h of incubation at 0.5 μM and 1 μM concentrations, respectively (Figure 6b,c). Western blot assays detected increased expression of Bax, Cleaved Caspase 3, Cleaved Caspase 9, and Cleaved PARP after moscatilin treatment, while Bcl-2 expression was inhibited, confirming the occurrence of apoptosis (Figure 6d,e).

### 2.5. Moscatilin Induces Apoptosis through MAPK Signaling Pathway

**Figure 7:**
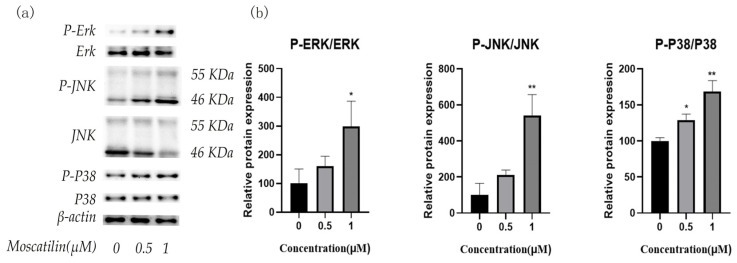
Moscatilin induces apoptosis through the MAPK signaling pathway. (**a**) Western blot assays were used to detect the protein levels of ERK, P-ERK, JNK, P-JNK, P38, and P-P38, and *β*-actin was used as a loading control. (**b**) The quantitative results were illustrated for Western blot. * *p* < 0.05, ** *p* < 0.01 compared with the 0 μM group.

The results of the western blot assay showed that moscatilin, at different concentrations, significantly promoted the phosphorylation of ERK, JNK, and P38 MAPK compared with the control group (0 μM group), suggesting that moscatilin may induce apoptosis in OSC-19 cells by promoting the phosphorylation of ERK, JNK, and P38 MAPK (Figure 7).

## 3. Materials and Methods

### 3.1. Reagents and Materials

3-(4,5-Dimethyl-2-thiazolyl)-2,5-diphenyl-2*H*-tetrazolium bromide (MTT) was purchased from Beyotime. Annexin V-FITC apoptosis detection kit was obtained from Beyotime. The antibodies used in Western blot analysis, which included *β*-actin rabbit monoclonal antibody (1:1000), JNK1 + JNK2 + JNK3 rabbit monoclonal antibody (1:1000), phospho-JNK1/JNK2/JNK3 (Thr183/Thr183/Thr221) rabbit monoclonal antibody (1:1000), ERK1/2 rabbit monoclonal antibody (1:1000), phospho-Erk1 (Thr202/Tyr204)/Erk2 (Thr185/Tyr187) rabbit monoclonal antibody (1:1000), P38 MAPK rabbit monoclonal antibody (1:1000), phospho-P38 MAPK (Thr180/Tyr182) rabbit polyclonal antibody (1:1000), MMP2 rabbit polyclonal antibody (1:1000), MMP9 rabbit polyclonal antibody (1:1000), PARP1 rabbit polyclonal antibody (1:1000), Caspase-9 rabbit monoclonal antibody (1:1000), Caspase 3 rabbit polyclonal antibody (1:1000), Bax rabbit polyclonal antibody (1:1000), Bcl2 rabbit polyclonal antibody (1:1000), were obtained from Beyotime. Anti-rabbit (Beyotime) was used for the secondary antibody.

### 3.2. Plant Material

*Dendrobium nobile* Lindl was harvested in Guizhou Province, China, in 2014 and identified as *Dendrobium nobile* Lindl by associate Prof. Yang Jian-Wen (Zunyi Medical University). A voucher specimen (No. 20141011) was deposited at the School of Pharmacy, Zunyi Medical University.

### 3.3. Extraction and Purification of Compounds ***1***–***6***

The dried *D. nobile* was crushed, then heated and refluxed with 90% methanol for extraction thrice, each time for 3 h. The methanol extract was evaporated under reduced pressure to obtain a crude extract, which was further suspended in water and extracted with EtOAc and *n*-BuOH (3 times each). EtOAc extract was subjected to silica gel column chromatography (70 mm × 660 mm, 300–400 mesh), elution with petroleum ether, EtOAc and methanol, and monitored using TLC to compile the resulted similar fractions. Based on TLC analysis, 12 fractions were further investigated.

Fr.3 was chromatographed over the Sephadex LH-20 column to afford 27 subfractions (Fr.3.1–Fr.3.27). Fr.3.7 was separated using semi-preparative HPLC with acetonitrile-H_2_O (65:35, *v*/*v*) at a flow rate of 3.5 mL/min to obtain 5 subfractions (Fr.3.7.1–Fr.3.7.5). Compound **3** was obtained from Fr.3.7.1 using recrystallization. Compound **6** was obtained from Fr.3.7.3 using recrystallization. Fr.7 was subjected to silica gel column chromatography (60 mm × 53 mm, 20 g, 300–400 mesh), eluted with a gradient of dichloromethane-methanol (20:1 *v*/*v*, 2100 mL, 15:1 *v*/*v*, 1500 mL, 10:1 *v*/*v*, 1500 mL, 5:1 *v*/*v*, 1500 mL). 

Then, by analyzing the TLC spots, Fr.2 was divided into eight subfractions (Fr.7.1–Fr.7.8). Fr.7.1 was chromatographed over a Sephadex LH-20 column to afford 26 subfractions (Fr.7.1.1–Fr.7.1.26). Fr.7.1.7 was separated using semi-preparative HPLC with methanol-H_2_O (62:38, *v*/*v*) at a flow rate of 3.5 mL/min to obtain compound **5**. Fr.7.2 was chromatographed over a Sephadex LH-20 column to afford 41 subfractions (Fr.7.2.1–Fr.7.2.41). Fr.7.2.7 was separated using semi-preparative HPLC with methanol-H_2_O (73:27, *v*/*v*, 10 min, 100:0, *v*/*v*, 3 min) at a flow rate of 3.0 mL/min to obtain two subfractions (Fr.7.2.7.1–Fr.7.2.7.2). Fr.7.2.7.1 was separated using HPLC with methanol-H_2_O (50:50, *v*/*v*,) at a flow rate of 3.0 mL/min to obtain compound **4**. Fr.7.4 was chromatographed over a Sephadex LH-20 column to afford 27 subfractions (Fr.7.4.1–Fr.7.4.27). Fr.7.4.22 was separated using semi-preparative HPLC with methanol-H_2_O (62:38, *v*/*v*, 73:27, *v*/*v*, 10 min, 100:0, *v*/*v*, 3 min) at a flow rate of 4.0 mL/min to obtain compounds **1** and **2**.

### 3.4. Cell Viability Assay

A cell viability assay was performed as described previously [22]. OSC-19 cells were plated on 96-well culture plates at a density of 3 × 10^3^ cells per well. After 12 h, cells were treated with varying concentrations (2.5 μM, 5 μM, 10 μM, and 20 μM) of moscatilin for 48 h. After treatment, cells were incubated with MTT (5 mg/mL) for 4 h. Carefully remove the medium and add 150 μL of DMSO to each sample and shake for 30 min. The cell absorbance value (A value) at 490 nm was detected using a micro plate reader. The results were analyzed using GraphPad Prism 8 software. 

### 3.5. Migration Assay

A migration assay was carried out as described previously [23]. The OSC-19 cells were cultured in a 6-well plate containing DMEM medium and 10% FBS. After confluence, wounds were performed in each well. Aspirate the supernatant, wash twice with PBS to remove cell debris, and add 1 mL of 0.5 μM and 1 μM moscatilin. Photos were taken using an inverted microscope at 0 h and 24 h, recording the scratch healing, and calculating the scratch healing area.

### 3.6. Apoptosis Assessment by AO/PI Staining

To determine the effect of moscatilin on OSC-19 cell viability, an AO/PI staining experiment was conducted [23]. OSC-19 cells were seeded into 24-well plates and incubated at 37 °C for 12 h. The cells were then treated with varying concentrations of moscatilin (0.5 μM and 1 μM) for 24 h. After removing the medium and washing twice with PBS, acridine orange (AO) and propidium iodide (PI) were added to the 24-well plate at a concentration of 100 μg/mL each and stained for 5 min at room temperature in the dark. Finally, the staining was observed using a fluorescence microscope. AO dye stains all nucleated cells, both live and dead, producing green fluorescence, while PI dye stains only dead nucleated cells, producing red fluorescence.

### 3.7. Annexin V-FITC/PI Assay for Apoptosis

The annexin-V FITC apoptosis detection kit was used to verify the moscatilin-induced cell death pattern using annexin-V FITC analysis [24]. Similarly, The OSC-19 cells were seeded into 6-well plates and treated with varying concentrations of moscatilin (0.5 μM and 1 μM) for 24 h, the samples were harvested. Then, 195 µL of annexinV-FITC binding buffer was used to resuspend the cells and stained with 10 µL PI and 5 µL annexin V-FITC for 15 min in the dark, and the apoptosis ratio was analyzed using flow cytometry. Each sample was measured in three separate replicate experiments.

### 3.8. Western Blot Analysis

Western blot analysis was performed as described previously [25]. OSC-19 cells were seeded into 6-well plates at a density of 4 × 10^5^ cells/well and incubated with varying concentrations of moscatilin (0.5 μM and 1 μM). Then cells were lysed in radioimmunoprecipitation a pssay buffer (RIPA), and protein concentration was quantified using a BCA protein assay kit. For western blot, proteins were separated in 10% SDS polyacrylamide gels and transferred to PVDF membranes. After blocking with 5% fat-free milk for 60 min at room temperature, they were incubated with primary antibodies at 4 °C overnight. The membranes were then washed with TBST buffer and incubated with an HRP-conjugated secondary antibody at room temperature for 60 min. After several washes of TBST, the blots were developed using enhanced chemiluminescence (ECL) solution.

### 3.9. Statistical Analysis

Statistical analyses were performed by using the GraphPad Prism 8 package. All experiments were carried out in triplicate and the averages of the three independent experiments were used as the statistical result. The results were represented as the mean ± standard deviation (SD). A one-way analysis of variance (ANOVA) was conducted, followed by Dunnett’s test compared with the control group. *p* ≤ 0.05 was considered statistically significant.

## 4. Conclusions

In summary, two new phenanthrene derivatives containing a spiro-lactone ring (**1** and **2**) and four known compounds (**3**–**6**) were isolated from *D. nobile*. The MTT assay showed that moscatilin had better inhibitory activity against OSC-19 human tongue squamous cells, prompting further investigation of its anti-cancer mechanism. The results revealed that moscatilin induced apoptosis in OSC-19 cells by regulating Bcl-2 family proteins and caspase family proteins. In addition, treatment with moscatilin increased the phosphorylation of JNK MAPK and P38 MAPK. Furthermore, moscatilin inhibited OSC-19 cell migration by suppressing EMT through the downregulation of MMP2 and MMP9 activity.

## Figures and Tables

**Table 1:** ^1^H and ^13^C (400/100 MHz) NMR data of compounds **1** and **2** in acetone-*d*_6_ (*δ* in ppm, *J* in Hz).

No.		1		2
	*δ*_C_	*δ*_H_	*δ*_C_	*δ*_H_
1	112.1		114.4	
2	158.6		159.0	
3	95.3	7.01, s	97.9	7.22, overlapped
4	152.3		152.1	
4a	113.8		118.3	
4b	127.1		125.7	
5	130.8	9.68, d, 9.4	130.7	9.69, d, 9.2
6	118.3	7.24, dd. 9.4, 2.8	118.1	7.22, overlapped
7	155.9		156.0	
8	106.9	7.68, d, 2.8	112.5	7.22, overlapped
8a	128.2		134.3	
9	153.7		130.6	7.50, d, 9.0
10	100.3	6.40, s	121.2	7.00, d, 9.0
10a	131.1		129.6	
1′	124.6		123.9	
2′	156.8		157.1	
3′	103.7	6.39, s	103.7	
4′	160.9		162.0	
5′	106.4	7.66, d, 2.6	106.5	7.67, br. s
6′	157.2		157.1	
7′	122.2	7.45, dd, 9.0, 2.6	122.2	7.45, br. d, 9.0
8′	126.7	9.11, d, 9.0	126.6	9.12, d, 9.0
9′	125.9		125.8	
10′	128.1		128.4	
11′	179.3		179.3	
12′	54.1		54.0	
13′	47.9	3.23, d, 18.5; 3.36, d, 18.5	48.7	3.32, d, 18.5; 3.39, d, 18.5
14′	201.1		201.0	

## Data Availability

All relevant data have been provided within the manuscript.

## Supplementary Materials

The following supporting information can be downloaded at: https://www.mdpi.com/article/10.3390/molecules28083423/s1, Figures S1–S14. ^1^H-NMR, ^13^C-NMR, ^1^H-^1^H COSY, HSQC, HMBC, NOESY, and HR-ESI-MS spectra of compounds **1** and **2**.

## Sample Availability

Samples of the compounds are available from the authors upon request.
