# Supplementary material for: Secondary Metabolites from Dendrobium nobile and Their Activities Induce Metabolites Apoptosis in OSC-19 Cells
Source: Molecules. 2023 Apr 13;28(8):3423. doi: 10.3390/molecules28083423 (PMC10145760; doi:10.3390/molecules28083423)
Supplement: Supplementary file 1 [file molecules-28-03423-s001.zip › molecules-2313006-supplementary.pdf]

Supporting information for

Secondary Metabolites from *Dendrobium Nobile* and Their Activities Induces Apoptosis in OSC-19 Cells

Yufan Meng [1, †], Maosheng Zhang [1, †], Yike Fang [1], Jianwen Yang [1], Minjian Dong [1, 2], Chengxin Sun [1, 2, *], Shiji Xiao [1, 2, *]

1 Key Laboratory of Basic Pharmacology of Guizhou Province and School of Pharmacy, Zunyi Medical University, Zunyi, Guizhou, 563000
2 Key Laboratory of Basic Pharmacology of Ministry of Education and Joint International Research Laborator Corresponding authors y of Ethnomedicine of Ministry of Education, Zunyi Medical University, Zunyi 563000, China
* Correspondence: E-mail address: suncx926@nenu.edu.cn; xiaoshiji84@163.com
† These authors contributed equally to this work.

Tables of Contents

S1. ^{1}H-NMR spectrum (400 MHz, acetone-d_6) of compound **1**

S2. ^{13}C-NMR spectrum (100 MHz, acetone-d_6) of compound **1**

S3. ^{1}H-^{1}H COSY NMR spectrum (400 MHz, acetone-d_6) of compound **1**

S4. ^{1}H-^{13}C HSQC NMR spectrum (acetone-d_6) of compound **1**

S5. ^{1}H-^{13}C HMBC NMR spectrum (acetone-d_6) of compound **1**

S6. NOESY spectrum (acetone-d_6) of compound **1**

S7. HR-ESI-MS of compound **1**

S8. ^{1}H-NMR spectrum (400 MHz, acetone-d_6) of compound **2**

S9. ^{13}C-NMR spectrum (100 MHz, acetone-d_6) of compound **2**

S10. ^{1}H-^{1}H COSY NMR spectrum (400 MHz, acetone-d_6) of compound **2**

S11. ^{1}H-^{13}C HSQC NMR spectrum (acetone-d_6) of compound **2**

S12. ^{1}H-^{13}C HMBC NMR spectrum (acetone-d_6) of compound **2**

S13. NOESY spectrum (acetone-d_6) of compound **2**

S14. HR-ESI-MS of compound **2**

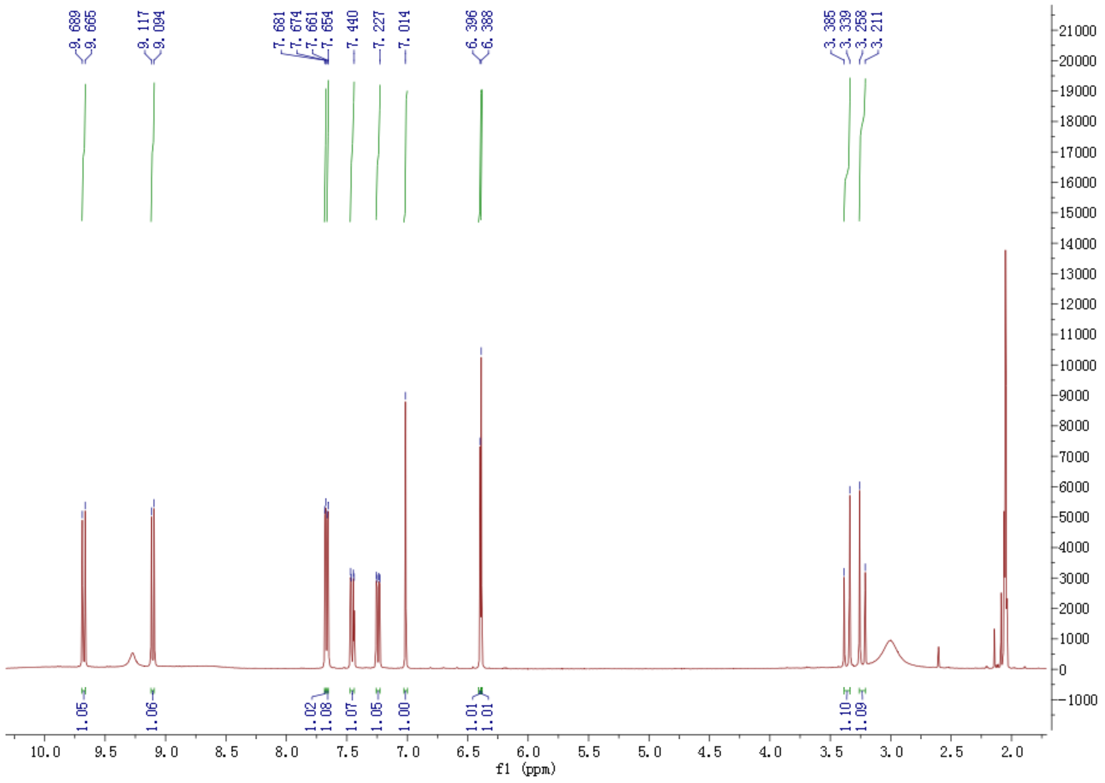

Figure S1. ^{1}H-NMR spectrum (400 MHz, acetone-d_6) of compound **1**

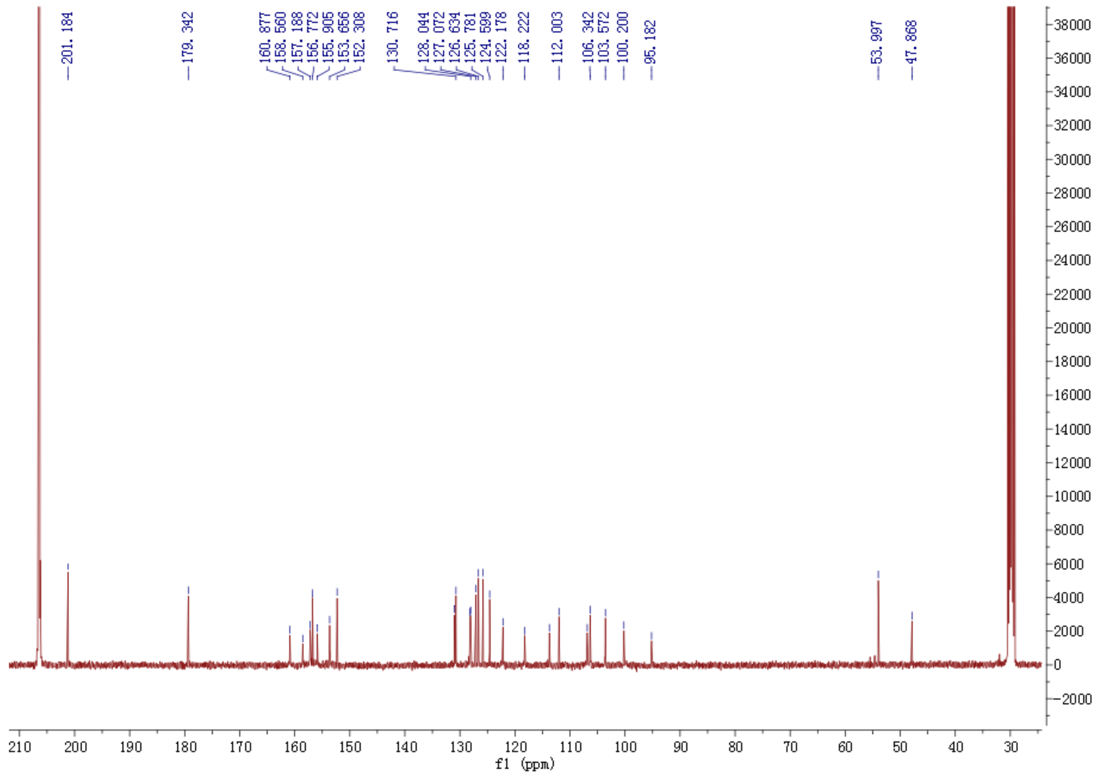

Figure S2. ^{13}C-NMR spectrum (100 MHz, acetone-d_6) of compound **1**

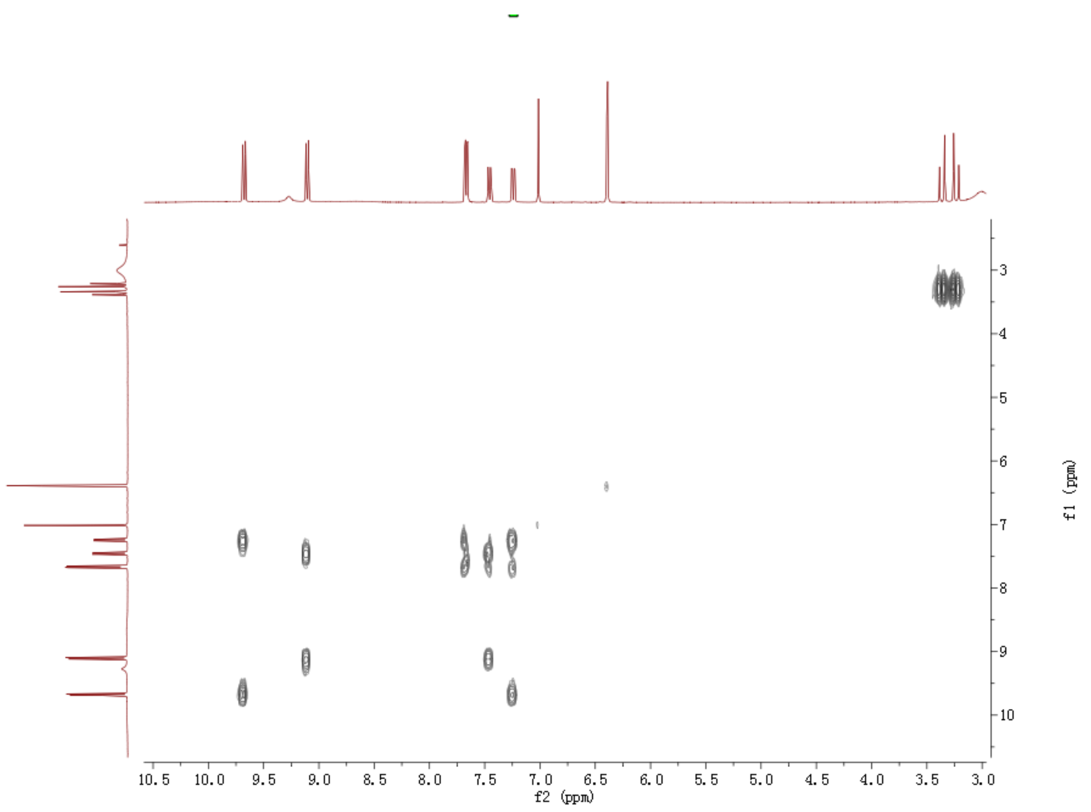

Figure S3. ^{1}H-^{1}H COSY NMR spectrum (400 MHz, acetone-d_6) of compound **1**

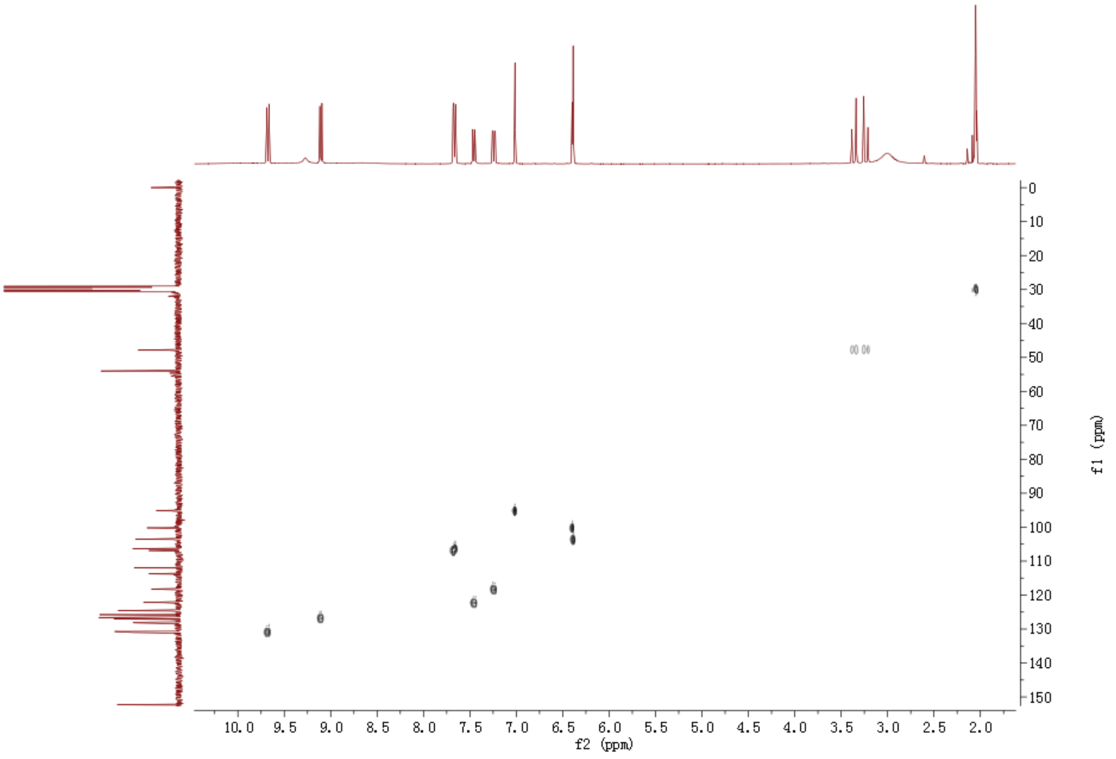

Figure S4. ^{1}H-^{13}C HSQC NMR spectrum (acetone-d_6) of compound **1**

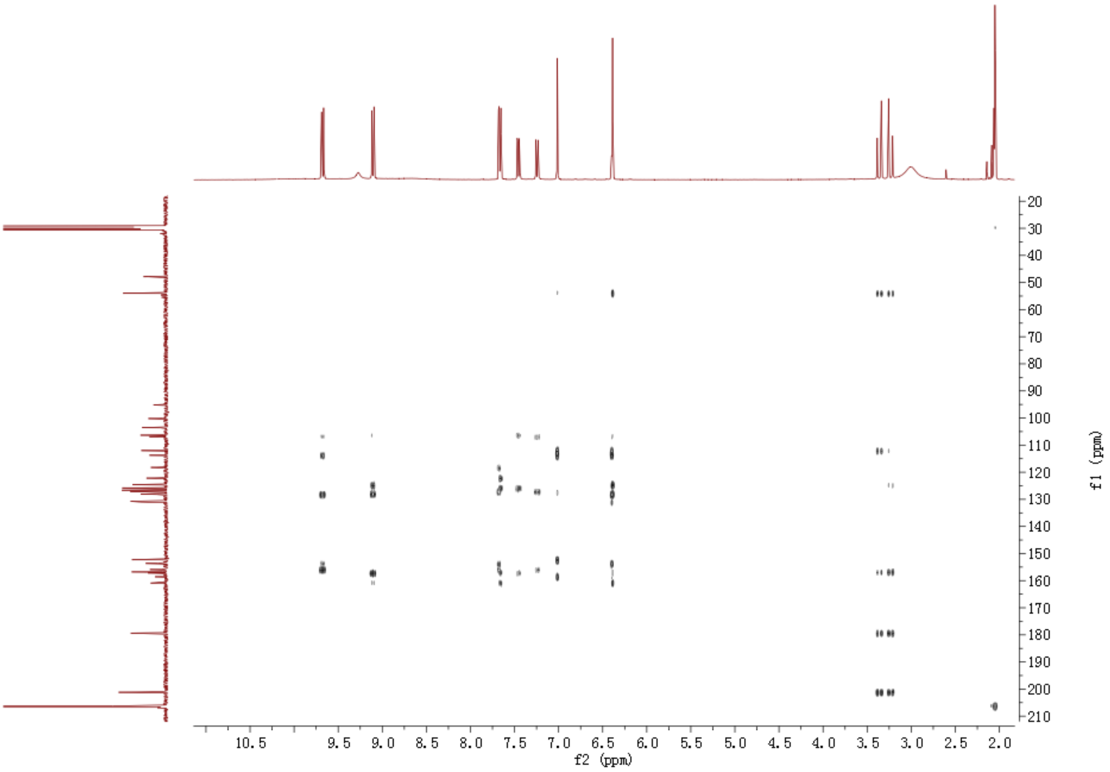

Figure S5. ^{1}H-^{13}C HMBC NMR spectrum (acetone-d_6) of compound **1**

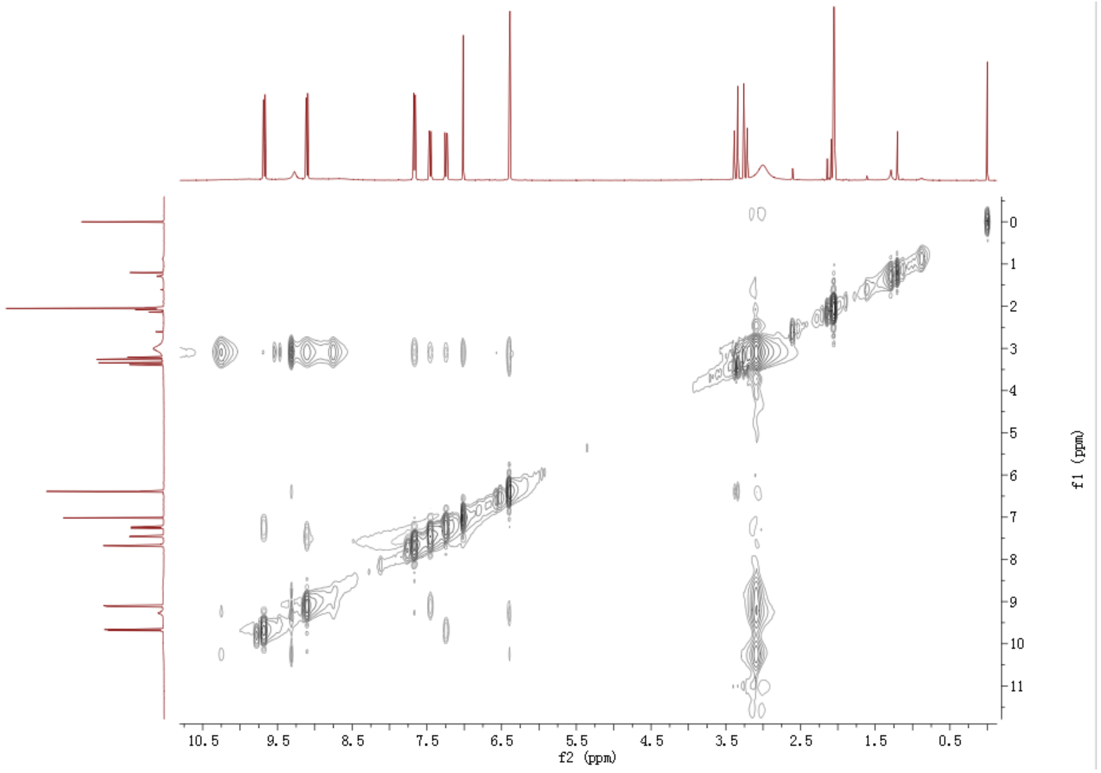

Figure S6. NOESY spectrum (acetone-d_6) of compound **1**

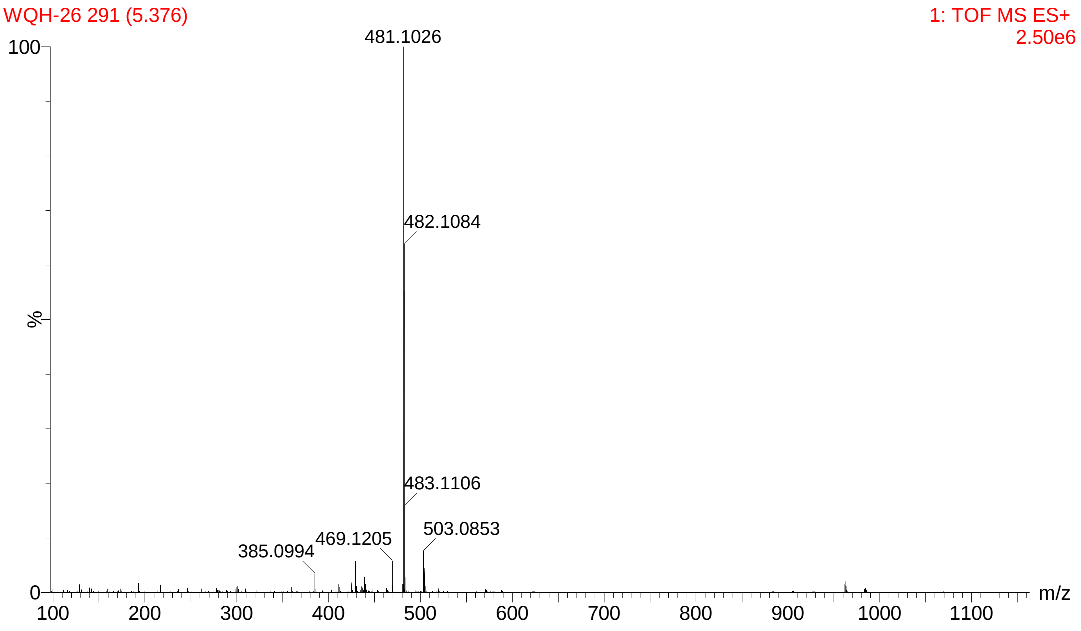

Figure S7. HR-ESI-MS of compound **1**

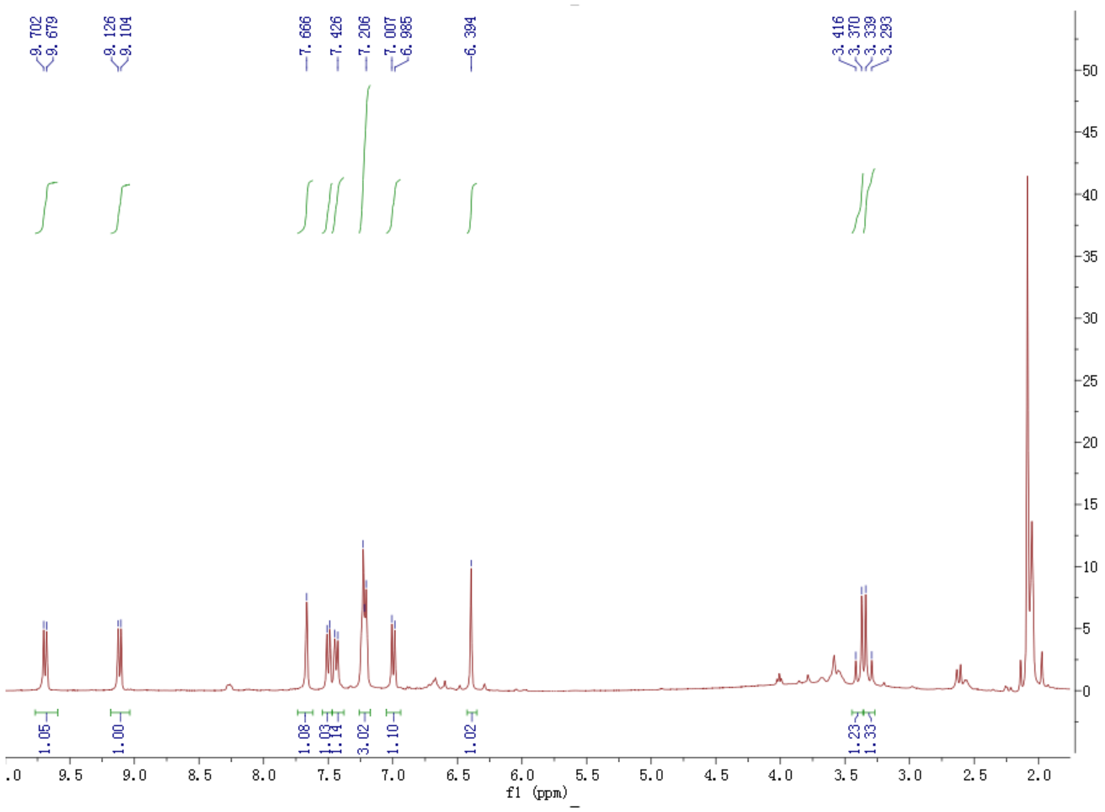

Figure S8. ^{1}H-NMR spectrum (400 MHz, acetone-d_6) of compound **2**

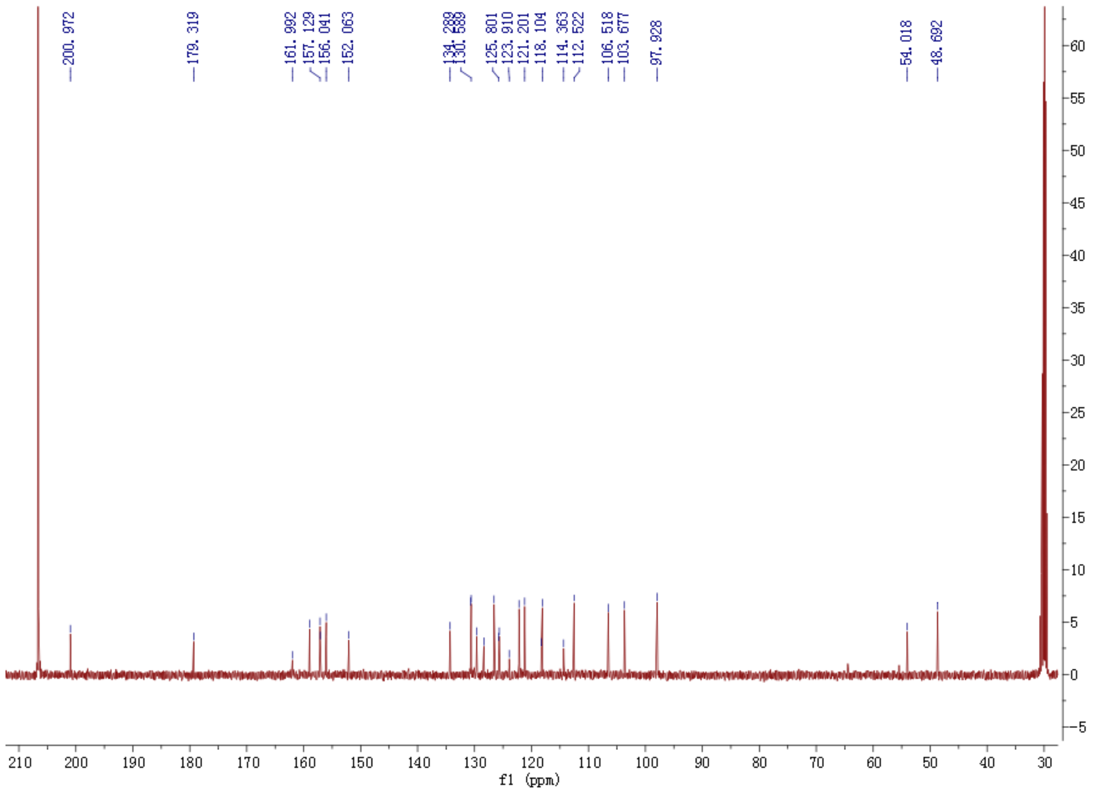

Figure S9. ^{13}C-NMR spectrum (100 MHz, acetone-d_6) of compound **2**

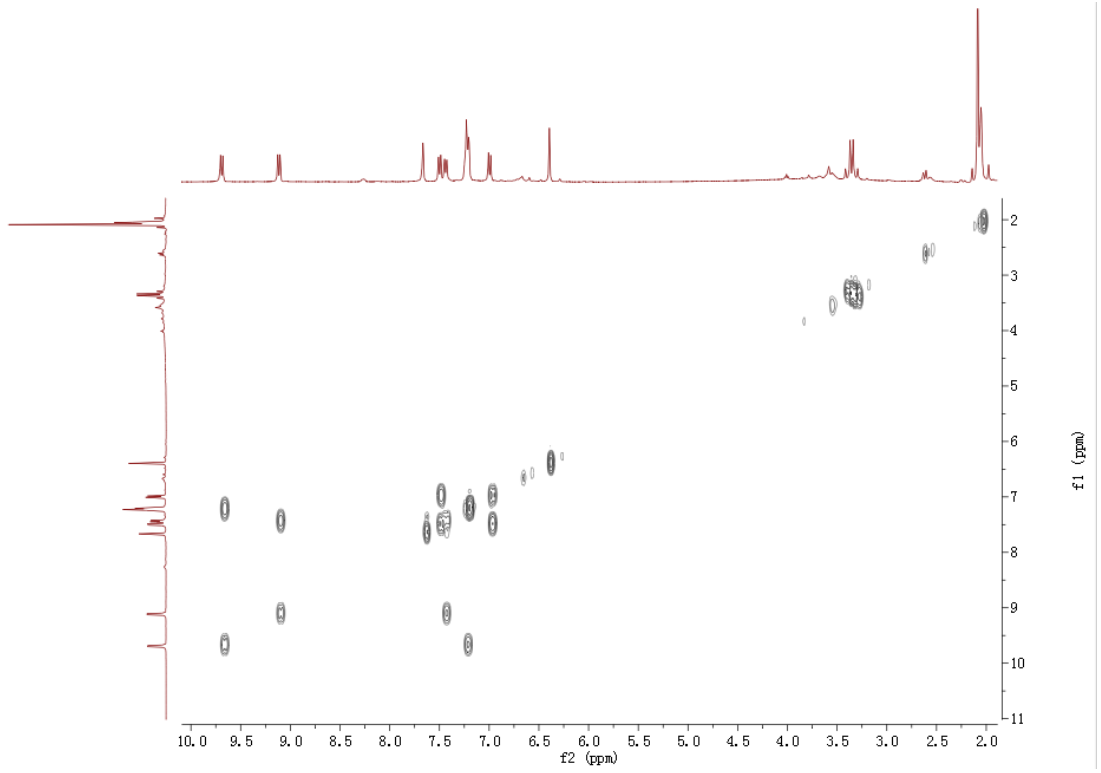

Figure S10. ^{1}H-^{1}H COSY NMR spectrum (400 MHz, acetone-d_6) of compound **2**

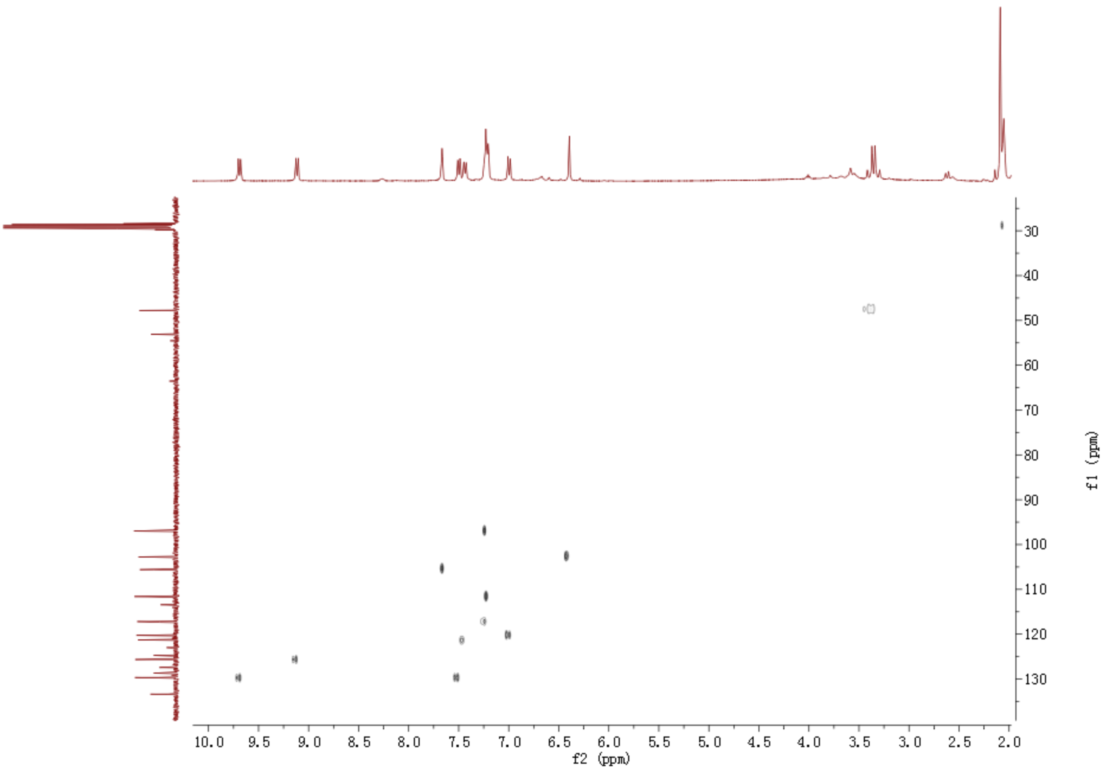

Figure S11. ^{1}H-^{13}C HSQC NMR spectrum (acetone-d_6) of compound **2**

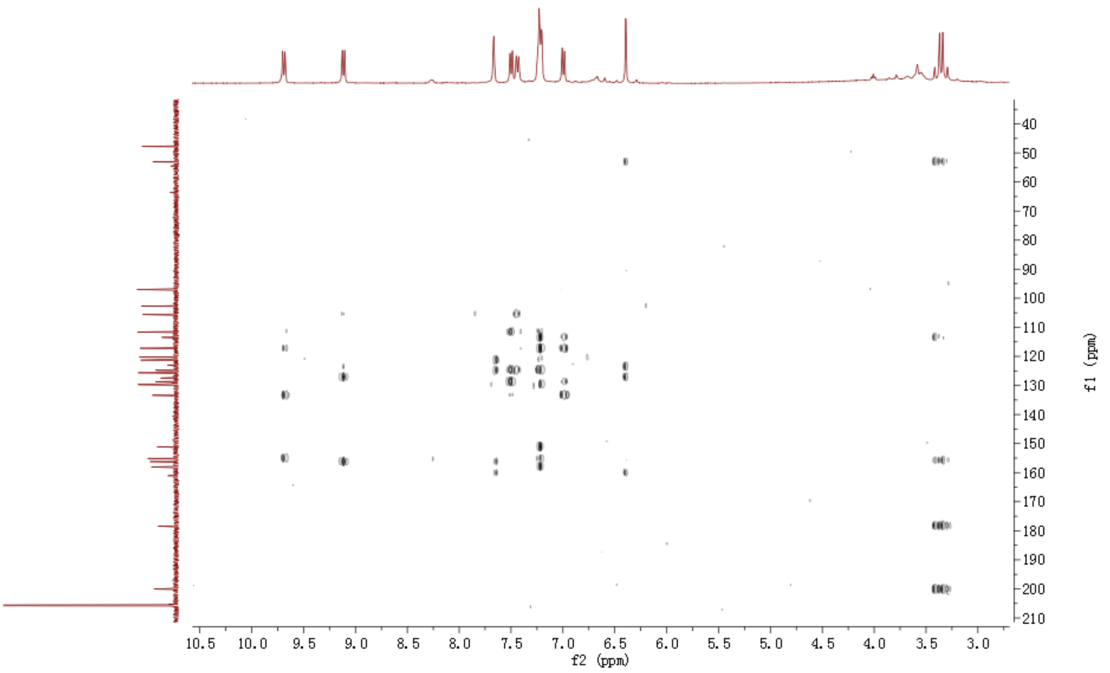

Figure S12. ^{1}H-^{13}C HMBC NMR spectrum (acetone-d_6) of compound **2**

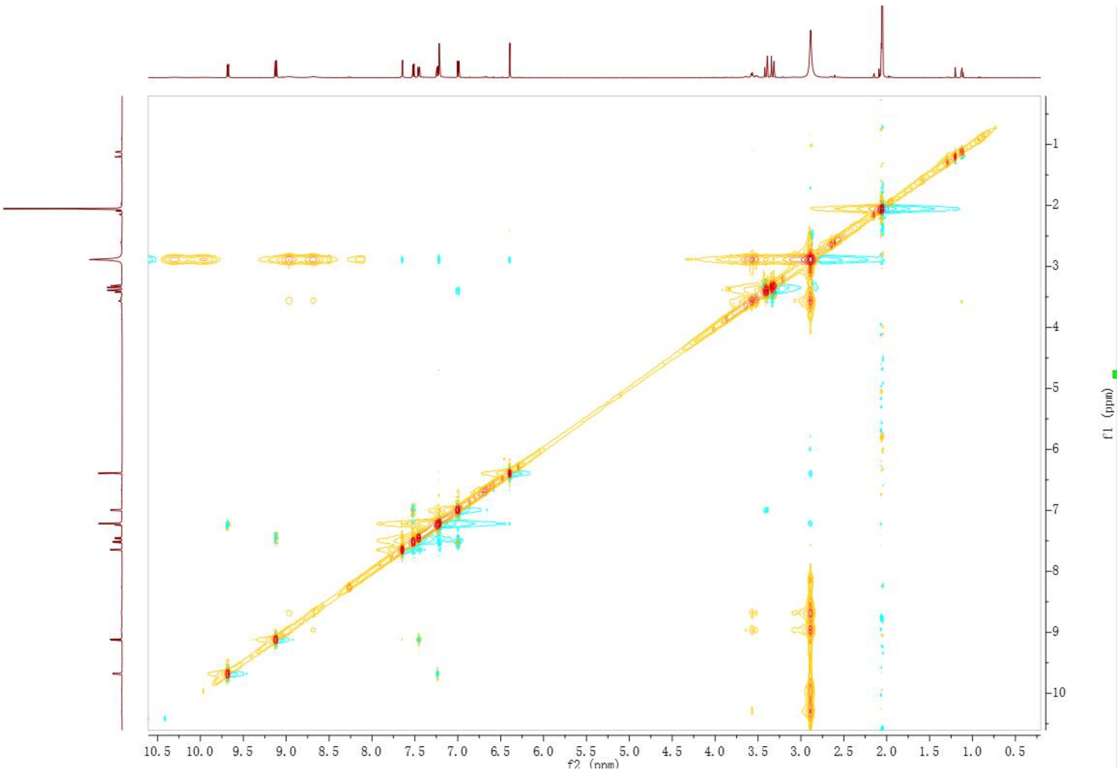

Figure S13. NOESY spectrum (acetone-d_6) of compound **2**

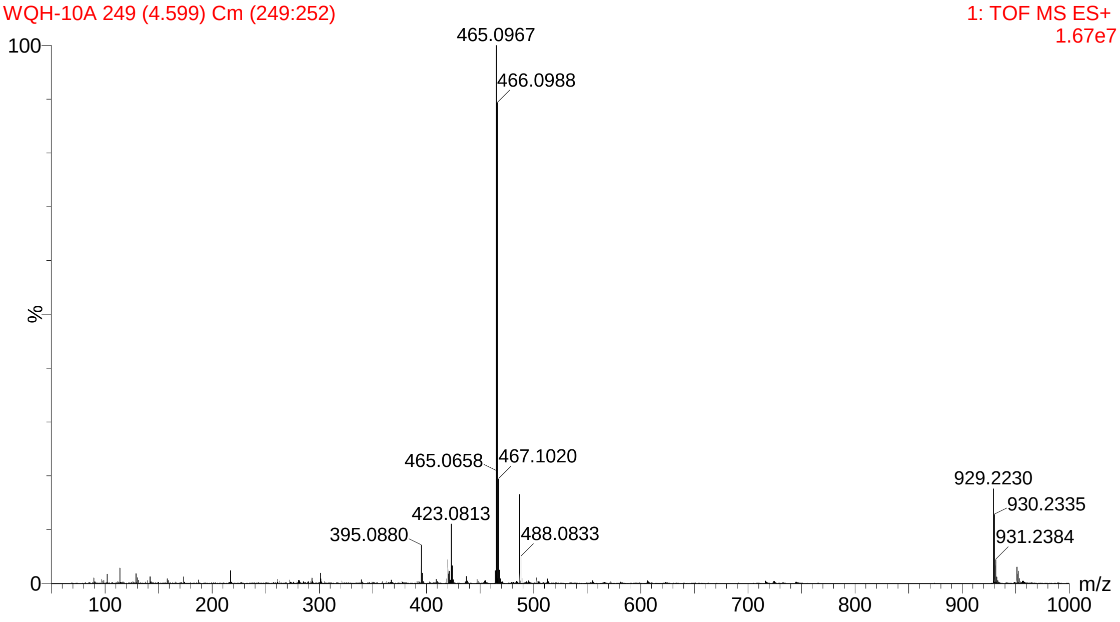

Figure S14. HR-ESI-MS of compound **2**
